# Silencing of Cytochrome P450 in Spodoptera frugiperda (Lepidoptera: Noctuidae) by RNA Interference Enhances Susceptibility to Chlorantraniliprole

**DOI:** 10.1093/jisesa/ieaa047

**Published:** 2020-06-02

**Authors:** Zhang Bai-Zhong, Su Xu, Zhen Cong-Ai, Lu Liu-Yang, Li Ya-She, Ge Xing, Chen Dong-Mei, Pei Zhang, Shi MIng-Wang, Chen Xi-Ling

**Affiliations:** 1 College of Resources and Environment, Henan Institute of Science and Technology, Xinxiang, Henan Province, P.R. China; 2 Department of Entomology, China Agricultural University, Beijing, P.R. China

**Keywords:** *Spodoptera frugiperda*, cytochrome (P450), insecticide detoxification, RNAi

## Abstract

Fall armyworm, *Spodoptera frugiperda* (Smith), has caused significant losses for crop production in China. The fall armyworm is mainly controlled by the chemical insecticides, whereas the frequent application of insecticides would result in the resistance development. Insect cytochrome P450 monooxygenases play an essential part in the detoxification of insecticides. In this study, five P450 genes were selected to determine the role in response to insecticides by RNA interference (RNAi). Developmental expression pattern analysis revealed that *S. frugiperda CYP321A8*, *CYP321A9*, and *CYP321B1* were highest in second-instar larvae among developmental stages, with 2.04-, 3.39-, and 8.58-fold compared with eggs, whereas *CYP337B5* and *CYP6AE44* were highest in adult stage, with 16.3- and 10.6-fold in comparison of eggs, respectively. Tissue-specific expression pattern analysis exhibited that *CYP321A8*, *CYP321B1*, and *CYP6AE44* were highest in the midguts, with 3.56-, 3.33-, and 3.04-fold compared with heads, whereas *CYP321A9* and *CYP337B5* were highest in wings, with 3.07- and 3.36-fold compared with heads, respectively. RNAi was also conducted to explore detoxification effects of the five P450 genes on chlorantraniliprole. The second-instar larvae became more sensitive to chlorantraniliprole with a higher mortality rate than the control, after silencing *CYP321A8*, *CYP321A9*, and *CYP321B1*, respectively. These findings strongly supported our viewpoint that *CYP321A8*, *CYP321A9*, and *CYP321B1* may play a critical role in insecticide detoxification. It will provide a basis for further study on regulation of P450 genes and the management of *S. frugiperda*.

Fall armyworm, *Spodoptera frugiperda* (Smith), is an invasive omnivorous pest throughout the world, mainly harming many crops such as corn, rice, sorghum, and peanut (Goergen et al. 2016, Liu et al. 2019). Chemical control is the most effective strategy to management of fall armyworm (Hardke et al. 2011, Okuma et al. 2018, Togola et al. 2018). Zhao et al. (2019) found that insecticides including chlorantraniliprole, spinetoram, emamectin benzoate, and so on still had a high activity to fall armyworm by determining the toxicity of 21 common chemical insecticides to the second-instar larvae of fall armyworm. However, the long-term use in large quantities of chemical insecticides could easily lead to resistance of pests (Al-Sarar et al. 2006, Burtet et al. 2017, Li et al. 2019).

Chlorantraniliprole, emamectin benzoate, spinetoram, and *Bacillus thuringiensis* were used as the main insecticides to control lepidoptera pests (Pan 2005, Cordova et al. 2006, Li et al. 2019). Some pests such as *Plutella xylostella* developed serious resistance to chlorantraniliprole (Hu et al. 2012, Wang et al. 2013, Xia et al. 2013), *Mythimna separate* in the field population developed certain resistance to chlorantraniliprole and emamectin benzoate (Dong et al. 2014, Liu et al. 2016), and fall armyworm also has developed resistance to *Bacillus thuringiensis* (Monnerat et al. 2015).

It has been reported that insecticides transformed into nontoxic or less toxic by the increased metabolic activity of detoxifying enzymes, which was one of the critical mechanisms for pest resistance against insecticides (Francis et al. 2006, Qiu 2014, Li et al. 2015). Detoxification metabolism and resistance to insecticides were affected by changing detoxification enzyme activity and related gene expression in pests (Chen et al. 2015, Elzaki et al. 2015). Cytochrome P450 is one of the critical metabolic enzyme systems in insects, which could metabolize a variety of endogenous and exogenous compounds (Zhu et al. 2010, Riveron et al. 2013, Edi et al. 2014). In addition, the increase of the activity of P450 induced by insecticides promoted the development of resistance and led pests to adapt to environments (van Pottelberge et al. 2008, Guo et al. 2012, Yang et al. 2015). Numerous P450 genes were frequently identified to be associated with insecticide resistance via the detoxification metabolism and usually belonged to the CYP4, CYP6, CYP9, and CYP12 families (Li et al. 2007, 2018; Hu et al. 2014). For example, *CYP6CM1* was overexpressed in the imidacloprid resistant whitefly *Bemisia tabaci* (Karunker et al. 2008). *CYP6BG1* was most possibly correlated with chlorantraniliprole resistance in *P. xylostella* (L.) (Li et al. 2018).

The inducible expression of some P450 genes by a variety of compounds was a common phenomenon of insect in exposure to various environment, which was called as induction. For example, the transcript expressions of some P450 genes were able to be induced by insecticides in the mosquito *Aedes aegypti* (Poupardin et al. 2010), the fruit fly *Drosophila melanogaster* (Fuchs et al. 1994, Baek et al. 2010), and *P. xylostella* (Bautista et al. 2007). The induction of detoxification genes was considered to be involved in insecticide resistance (Terriere 1984, Scharf et al. 2001, Goff et al. 2006). Hence, it is necessary to confirm the related detoxification genes in response to insecticide exposure. Insecticide at sublethal concentrations could affect the physiological behaviors of pests to further promote the development of insecticide resistance (Han et al. 2011, Zhang et al. 2019). Therefore, it is of great significance to study the induction effect of insecticides on fall armyworm for assessing its role in pest control.

In our previous study, we found that *CYP321A8*, *CYP321A9*, *CYP321B1*, *CYP337B5*, and *CYP6AE44* could be strongly induced by chlorantraniliprole (unpublished results, *P* < 0.05, *n* = 3). Next, tissue and developmental distribution of these P450 genes were determined by quantitative real-time PCR, and RNA interference was conducted to examine the role of P450 genes under the effect of insecticide. These results provided evidence for understanding the functions of P450 genes in the insecticide influence.

## Materials and Methods

### Insects

The strain of fall armyworm used in the experiment was provided by College of Plant Protection, Yunnan Agricultural University. The moth was raised in the cages with a temperature of 23–25°C, relative humidity of about 60–70%, and photoperiod of 16:8 (L:D) h.

The artificial diet for the larva contains corn flour, soybean powder, yeast extract powder, citric acid, vitamin C, sorbic acid, vitamin, erythromycin, propionic acid, and vitamin E. Three-day age adults were fed with the same artificial diet and also supplied with a 10% sugar solution. Oviposition was finished on the leaves of corn, and collected the eggs for the next generation. The fall armyworm was reared in a growth chamber set to the aforementioned environmental conditions with no exposure to any insecticides. The samples of developmental stages and tissues of *S. frugiperda* were collected as follows: eggs (*n* = 80), second-instar larvae (*n* = 5), pupae (*n* = 5) and mixed-sex 3-d age adults (*n* = 5), the different tissues were taken from 3-d age adults (*n* = 6), each sample was repeated three times. All samples were snapped in liquid nitrogen flash freezer and then stored at −80°C for RNA extraction.

### Chemicals

Chlorantraniliprole (98%) was taken from Shenzhen Noposion Agrochemicals Co., Ltd. (Shenzhen, China). Triton X-100 was supplied by Sigma–Aldrich Co. (Saint Louis, MO). All insecticide chemicals and solvents used were analytical grade. TRIzol reagent was obtained from Invitrogen (Shanghai, China); Agarose, DNase I, and SYBR Green I were purchased from the TaKaRa Company (Dalian, China); Taq DNA polymerase and DNA Marker DL 2000 were both purchased from the Sangon Company (Shanghai, China); and the MEGAscript RNAi kit was purchased from Ambion (Austin, TX).

### RNA Isolation and cDNA Synthesis

Total RNA was isolated by using the TRIzol kit (Invitrogen, Carlsbad, CA) according to the manufacturer’s instructions. Measure of concentration and quality of RNA were conducted by a NanoVue UV–Vis spectrophotometer (GE Healthcare Bio-Science, Uppsala, Sweden) at 260 nm (the optical density) and the A260/A280 (absorption ratio); RNA integrity was determined on 1% agarose gel using electrophoresis, and genomic DNA was erased with DNase I (TaKaRa, Madison, WI). First-strand cDNA synthesis was conducted with 1.0 μg of total RNA using the PrimeScript RT reagent kit with gDNA Eraser (Takara Biotechnology, Dalian, China), and stored at −20°C.

### Quantitative Real-Time PCR Analysis

The five overexpressed genes under chlorantraniliprole exposure were selected to determine their relative expression level by quantitative real-time PCR (qPCR) method at different developmental stages, within different tissues and dsRNA feeding of fall armyworm. Three replications were conducted for each sample. The designed primers for each specific genes were shown in Table 1. RNA was extracted as mentioned above, and 500-ng RNA with DNA-free was reverse-transcribed for synthesizing the first-strand cDNA using the PrimerScript RT Reagent Kit Perfect Real Time Kit (Takara, Dalian, China) based on the manufacturer’s instructions. The qPCR (20 µl) contained were performed in a mixture. The reaction was performed on an ABI 7500 Real Time PCR system (Applied Biosystems) with three biological replicates and two technical replications for each cDNA sample. *GADPH* was used as the reference gene for normalization of the expression levels (do Nascimento et al. 2015). The relative expression level for the selected target genes was calculated using the 2^−ΔΔCT^ method (Livak and Schmittgen 2001).

**Table 1.** The primer for qPCR

Gene name	Accession number	Sequence (5′–3′)	*T*_m_ (°C)	Product length
*CY321A8*	KC789751.1	F: cgctctttgtcatcgatccg	57.45	106
		R: tcggtcagttgatccccttc	57.45	
*CYP321A9*	KC789752.1	F: gagatagagcctacgaccgg	59.50	96
		R: caacgtcgcatagatcgcat	55.40	
*CYP321B1*	KC789754.1	F: gccatcgcgcatatcctaag	57.45	112
		R: cgtggaaccaactcgatgtc	57.45	
*CYP337B5*	KJ671580.1	F: gttcgtgtttgggaagcagt	55.40	123
		R: tcagggtgcttgagaaggag	57.45	
*CYP6AE44*	KJ671576.1	F: gtgtgaccgagttgccttac	57.45	145
		R: aaatgcacgcgaagtccttt	53.35	
*GADPH*	KC262638.1	F: cggtgtcttcacaaccacag	57.45	111
		R: ttgacaccaacgacgaacat	53.35	

### RNA Interference

#### dsRNA Synthesis

The specific primers including a T7 polymerase promoter sequence were designed to obtain the products of *CYP321A8*, *CYP321A9*, *CYP321B1*, *CYP337B5*, and *CYP6AE44* by real time PCR (RT–PCR). The detailed information of primers were listed in Table 2. The synthesis of dsRNAs was performed using the MEGAscriptRNAi kit (Ambion) with the products of RT–PCR as templates. Templates of dsRNA synthesis are from five fragments: 337 bp for *CYP321A8*, 400 bp for *CYP321A9,* 386 bp for *CYP321B1,* 400 bp for *CYP337B5*, and 397 bp for *CYP6AE44* according to the Transcript Aid T7 High Yield Transcription Kit (Termo Scientific, Wilmington, DE). A dsRNA specific for green fluorescent protein (GFP, ABE28520.1) was used as a negative control in the experiment. All synthesized dsRNAs were resuspended using RNase-free water and verified as a single band by agarose gel electrophoresis. The concentration of dsRNAs was determined using a NanoVue UV–Vis spectrophotometer (GE Healthcare Bio-Science, Uppsala, Sweden).

**Table 2.** The primers used in RNAi knockdown

Gene name	Accession number	Primer name	*T*_m_ (°C)	Sequence (5′–3′)	Product length (bp)
*GFP*	—	GFP(T7)–F	69.7	taatacgactcactatagggTGACCACCCTGACCTAC	288
		GFP(T7)–R	68.8	taatacgactcactatagggTTGATGCCGTTCTTCTGC	
*CYP321A8*	*KC789751.1*	*CYP321A8*(T7)–F	69.38	taatacgactcactatagggCGCTCTTTGTCATCGATCCG	337
		*CYP321A8*(T7)–R	68.35	taatacgactcactatagggTCTGACCCAATGCCGAAGAT	
*CYP321A9*	*KC789752.1*	*CYP321A9*(T7)–F	67.32	taatacgactcactatagggTTGTGGAGCTATCTTCGGCA	400
		*CYP321A9*(T7)–R	67.32	taatacgactcactatagggCAACGTCGCATAGATCGCAT	
*CYP321B1*	*KC789754.1*	*CYP321B1*(T7)–F	68.35	taatacgactcactatagggACGTACGATGCAGTCTTGGA	386
		*CYP321B1*(T7)–R	69.38	taatacgactcactatagggCGTACACCACCCTCCTTGAT	
*CYP337B5*	*KJ671580.1*	*CYP337B5*(T7)–F	67.32	taatacgactcactatagggGGTAGCCCCGTAAACCTTGT	400
		*CYP337B5*(T7)–R	67.32	taatacgactcactatagggGCTACAACCGGATGGAAGAC	
*CYP6AE44*	*KJ671576.1*	*CYP6AE44*(T7)–F	68.35	taatacgactcactatagggCCCGAATACATCAAGACCGT	397
		*CYP6AE44*(T7)–R	68.35	taatacgactcactatagggTCATTCTGGCAGTGTCGAAG	

#### In Vivo RNAi

Bioassay method of dsRNA artificial diet was prepared as described by Zhang et al (2016a), and the device used for dsRNA feeding assays was the same as described above. The *dsCYP321A8*, *dsCYP321A9*, *dsCYP321B1*, *dsCYP337B5*, and *dsCYP6AE44* were mixed into the artificial diets at 50 mg/kg (w/w) concentration, respectively. The ds*GFP* mixed into the artificial diet at 50 mg/kg (w/w) concentration was used as the control. Each sample was repeated three times.

To evaluate the effect of dsRNA knockdown on the target P450 genes expression, 50 healthy second-instar larvae were gently selected into the tube with a brush. Meanwhile, the tube was sealed with a piece of Chinese art paper by solid glue. The second-instar larvae were prepared to feed on this artificial diet with dsRNA for 36 h. The surviving larvae were collected and snapped using liquid nitrogen flash freezer and then stored at −80°C for RNA isolation. Each sample was repeated three times.

#### Sensibility to Chlorantraniliprole in Second-Instar Larvae after RNAi

To assess the role of P450 RNAi on the insecticide sensitivity of second-instar larvae, the leaf-dipping bioassay was carried out as described by Dong et al. (2014) with minor change. Insecticides were dissolved in acetone and then diluted to serial concentrations with 0.05% (v/v) Triton X-100 in water. Corn leaves were cut into pieces 20 mm long. The leaves were dipped into 0.05% (v/v) Triton X-100 water with insecticides for 10 s or dipped into 0.05% (v/v) Triton X-100 water without insecticides as a control. The leaves were put into glass petri dishes (20 cm in length, 60 mm diameter). Each concentration had three biological repeats, each with 20 healthy second-instar larvae. Mortality was recorded after insecticide exposure at 48 h. The larvae fed (*n* = 30) on dsRNA for 24 h were exposed to chlorantraniliprole at the lethal concentration (LC_50_), 1 mg/l chlorantraniliprole based on our previous experiments (unpublished results), then the mortality of dsRNA-fed larvae was observed after 24 h, each sample (survivals, *n* = 5) was repeated three times.

### Data Analysis

All the data analysis was conducted using the GraphPad InStat 3.0 software (GraphPad Software, San Diego, CA).

## Results

### Expression Profiling of the Five P450 Genes at Developmental Stages and Tissues

**Fig. 1.**
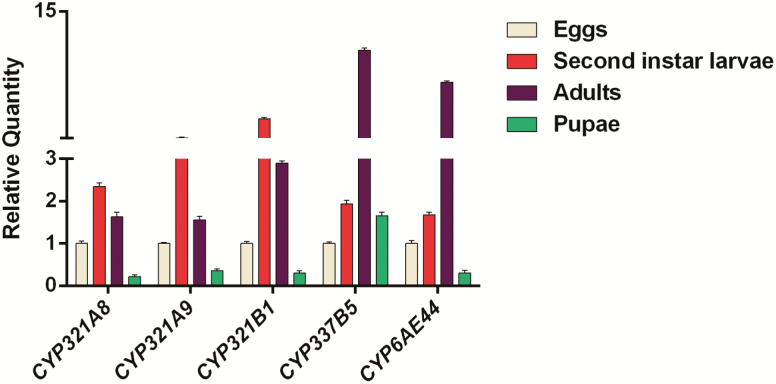
Developmental expression levels of the five P450 genes in *Spodoptera frugiperda*. Data are means ± SE of three biological replicates. The relative expression was calculated using the 2^−ΔΔCT^ method based on the value of the egg expression, which was ascribed an arbitrary value of 1. The bars with lowercase letters are significantly different according to the one-way ANOVA, followed by Tukey’s multiple comparison test (*P* < 0.05).

The expression profiling of the five P450 genes overexpressed under inseceticide exposure were further measured at various developmental stages and different tissues, respectively. These P450 genes, *CYP321A8*, *CYP321A9*, and *CYP321B1*, were the highest in second-instar larvae among developmental stages, with 2.35-, 3.03-, and 4.86-fold compared with the eggs (*P* < 0.05; *n* = 3), whereas *CYP337B5* and *CYP6AE44* were the highest at the adult stage, with 11.31- and 8.67-fold in comparison with eggs (*P* < 0.05; *n* = 3). In addition, *CYP321A8*, *CYP321A9*, *CYP321B1*, and *CYP6AE44* were lowest at the pupae stage, with 0.221-, 0.362-, 0.302-, and 0.308-fold of the expression level compared with egg stage (*P* < 0.05; *n* = 3; Fig. 1).

**Fig. 2.**
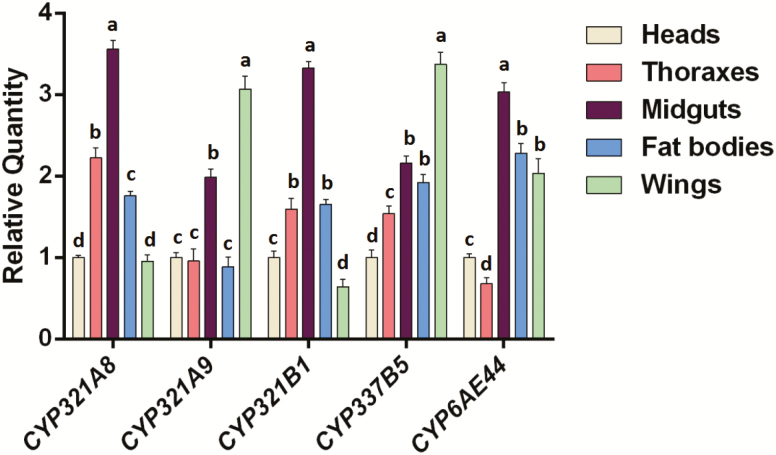
The tissue expression profiles of the five P450 genes in *Spodoptera frugiperda*. The relative expression was calculated using the 2^−ΔΔCT^ method based on the expression value of the adults head, which was assigned an arbitrary value of 1. The bars with lowercase letters are significantly different according to the one-way ANOVA, followed by Tukey’s multiple comparison test (*P* < 0.05).

For analyses of the tissue distribution expression level of these five P450 genes, the heads, thoraxes, midguts, fat bodies, and wings were detached from 3-d age adults. The results indicated that the expressed level of *CYP321A8*, *CYP321B1*, and *CYP6AE44* were the highest in the midguts, with 3.56-, 3.33-, and 3.04-fold compared with heads, whereas *CYP321A9* and *CYP337B5* were the highest in wings with 3.07- and 3.36-fold compared with heads (*P* < 0.05; *n* = 3; Fig. 2).

### Knockdown of the Five P450 Genes Enhances the Sensibility of Fall Armyworm to Chlorantraniliprole

**Fig. 3.**
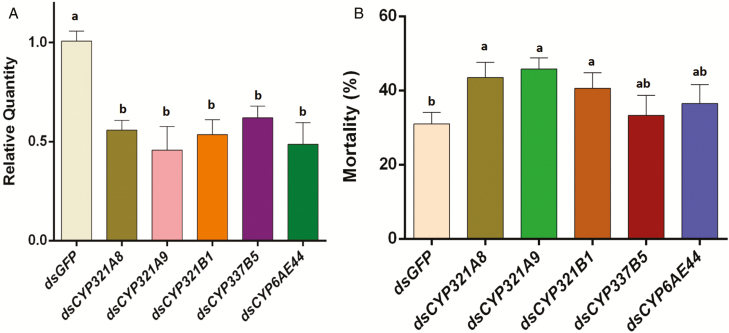
Relative expression levels of the target P450 gene and the mortality (%) of *Spodoptera frugiperda* at the 1 mg/l chlorantraniliprole. (A) Relative expression of the target P450 gene and (B) mortality (%) of *Spodoptera frugiperda* at 1 mg/l chlorantraniliprole. The data were expressed as the means ± SE. The bars with lowercase letters are significantly different according to the one-way ANOVA, followed by Tukey’s multiple comparison test (*P* < 0.05).

RNAi was conducted to study the five P450 genes and their effects on insecticide susceptibility. As a new second-instar larva, fall armyworm feeds dsRNA synchronously. To determine silencing efficiency of *CYP321A8*, *CYP321A9*, *CYP321B1*, *CYP337B5*, and *CYP6AE44*, qPCR was carried out in the suppression of RNAi experiments (Fig. 3A). The relative expression level of *CYP321A8*, *CYP321A9*, *CYP321B1*, *CYP337B5*, and *CYP6AE44* in ds*CYP321A8*-fed, ds*CYP321A9*-fed, ds*CYP321B1*-fed, ds*CYP337B5*-fed, and ds*CYP6AE44*-fed second-instar larvae were decreased compared with ds*GFP*-fed second-instar larvae at 36 h, respectively (*P* < 0.05; *n* = 3). The expression of the target P450 genes was significantly reduced by 44.2% (*CYP321A8*), 54.3% (*CYP321A9*), 46.4% (*CYP321B1*), 38.9% (*CYP337B5*), and 51.5% (*CYP6AE44*) compared with those ds*GFP*-fed second-instar larvae at 36 h (*P* < 0.05; *P* < 0.05; *n* = 3).

The sensitivity of *S. frugiperda* to chlorantraniliprole was investigated after silencing *CYP321A8*, *CYP321A9*, *CYP321B1*, *CYP337B5*, and *CYP6AE44*. The results revealed that knockdown of *CYP321A8*, *CYP321A9*, and *CYP321B1* increased the sensitivity to chlorantraniliprole with higher mortalities compared with controls (*P* < 0.05; *n* = 3). It was significantly higher in ds*CYP321A8*-fed, ds*CYP321A9*-fed, and ds*CYP321B1*-fed second-instar larvae (43.53, 45.79, and 40.62%, respectively) compared with controls (ds*GFP*-fed second-instar larvae; 31.01%) after RNAi at 24 h (*P* < 0.05; *n* = 3), then exposed to chlorantraniliprole for 24 h. While no significant difference of mortality was observed in ds*CYP321B1*-fed, and ds*CYP337B5*-fed and ds*CYP6AE44*-fed second-instar larvae (33.35 and 36.49%, respectively) compared with controls (ds*GFP*-fed second-instar larvae; 31.01%) after RNAi at 24 h, then exposed to chlorantraniliprole for 24 h (*P* < 0.05; *n* = 3).

## Discussion

The importance of P450 genes on the insecticide metabolism via detoxification or activation promoted a large number of research to focus on it (Pavek and Dvorak 2008, Scott 2008, Zhang et al. 2020). Inducibility by various compounds especially by insecticide was one common feature of multiple P450 genes, and this phenomenon was also found in our previous studies (Liu et al. 2011, Zhang et al. 2016a, b). Based on the P450 genes induction, five P450 genes, including *CYP321A8*, *CYP321B1*, *CYP321A9*, *CYP337B5*, and *CYP6AE44*, were proved to be induced by chlorantraniliprole in *S. frugiperda* in our previous study (unpublished results, *P* < 0.05, *n* = 3). These results were somewhat consistent with findings that some of *CYP321*, *CYP6*, and *CYP9* subfamiles could be induced by insecticides (Giraudo et al. 2015). In order to further explore the role of *CYP321A8*, *CYP321B1*, *CYP321A9*, *CYP337B5*, and *CYP6AE44* under the effect of chlorantraniliprole, tissue and developmental distribution of these P450 genes were determined by quantitative real-time PCR, and RNA interference was conducted to examine the role of P450 genes under the effect of chlorantraniliprole.

The results from chlorantraniliprole induction experiment exhibited that the transcripts of the five P450 genes were significantly induced in response to chlorantraniliprole with different degree (unpublished results). The expression profiles of these five P450 genes were further measured at the whole developmental stages, and in various tissues, especially the midgut and the fat body. Both tissues were thought to be important for the detoxification reaction in insects, and most detoxification P450s were found in these two tissues (Nikou et al. 2003). Other tissues such as the rain and nervous system were also found with the expression of P450 genes, which were associated with insecticide resistance (Liu and Scott 1998, Mao et al. 2009). Furthermore, cytochrome P450 genes were expressed at one/some specific developmental stages or tissue of animals, which may be correlated with the specific function (Chung et al. 2009).

In our present study, *CYP321A8*, *CYP321A9*, *CYP321B1*, and *CYP6AE44* were higher in the midgut, whereas *CYP321A9* and *CYP337B5* were highest in wings compared with the other tissues.

The observed higher expression of *CYP321A8*, *CYP321B1*, and *CYP6AE44* in the midgut might reflect a role for *CYP321A8*, *CYP321A9*, *CYP321B1*, and *CYP6AE44* in the metabolism of insecticides. These results were somewhat consistent with findings that these genes may be involved in mechanisms/detoxication or regulation of insecticides as the tissue-specific expression of genes was related to detoxification, which illustrated potentially their functions on the biology or physiology, and the midgut and fat body tissue of insects were always considered important as detoxification organs. In addition, the expression of these resistance-related genes was closely associated with the developmental period of the pests (Mao et al. 2009, Long et al. 2013). Our present results indicated that these P450 genes, *CYP321A8*, *CYP321A9*, and *CYP321B1*, were highest in the second-instar larvae with 2.04-, 3.39-, and 8.58-fold compared with those of the eggs, while *CYP337B5* and *CYP6AE44* were highest at adult stage, with 16.3- and 10.6-fold compared with those of eggs. *CYP321A8*, *CYP321A9*, and *CYP321B1*, and *CYP6AE44* were lowest in pupae stage, with 0.191-, 0.437-, 0.214-, and 0.276-fold compared with eggs. These results were somewhat consistent with the findings that these genes may be involved in mechanisms/detoxication or regulation of insecticides because of the larva as a more amenable in insecticide resistance. These findings strengthened the possibility that *CYP321A8*, *CYP321A9*, and *CYP321B1* may be significant for chlorantraniliprole resistance in fall armyworm.

In order to further clarify the function of the five P450 genes on the involvement in insecticide detoxification, we deemed the larvae as a more amenable in insecticide resistance for use in RNAi experiments, as previous studies into dsRNA acquisition by larvae have suggested that they can rapidly acquire dsRNA through a combination of feeding. Our study showed that oral delivery was capable of inducing RNAi in the second-instar larvae, as was also observed in Lepidoptera pests (Zhang et al. 2016a, Al Baki et al. 2020, Kunte et al. 2020). RNAi method supplied the possibility for new viewpoint of pest management. In the present study, the dsRNA knockdown results indicated that the efficency of dsRNA for silencing the target genes was highly ranging from 38.9 to 54.3% in comparison with the control using dsRNA feeding method. Our results were consistent with the verified findings (Zhang et al. 2016a, 2019; Ran et al. 2018).

Analysis of the knockdown effects of *CYP321A8*, *CYP321B1*, *CYP321A9*, *CYP337B5*, and *CYP6AE44* on the second-instar larvae susceptibility to chlorantraniliprole. Mortality was higher in the ds*CYP321A8*, ds*CYP321B1*, and ds*CYP321A9*-fed second-instar larvae than in the ds*GFP*-fed second-instar larvae under chlorantraniliprole treatment. These results indicated that *CYP321A8*, *CYP321B1*, and *CYP321A9* may be responsible for detoxification of insecticides, while some extra experiments must be carried out.

### Conclusion

Our results demonstrate that RNAi by feeding dsRNA is successful in second-instar larvae. Future studies should focus on demonstrating *CYP321A8*, *CYP321B1*, and *CYP321A9*, metabolism of chlorantraniliprole to determine a more precise definition of its role in detoxification. Knockdown of *CYP321A8*, *CYP321B1*, and *CYP321A9* in another developmental stage of the fall armyworm collected from the field is also under way to further support these views.
